# Transcriptomic analysis reveals FcγR-mediated phagocytosis as a key pathway for the anti-inflammatory action of *Polygonatum sibiricum* polysaccharides in loach

**DOI:** 10.3389/fgene.2026.1733253

**Published:** 2026-06-19

**Authors:** Yue Zhao, Xiangwei Xu, Ruike Fan, Lishang Dai, Linxinyi Chen

**Affiliations:** 1 School of Biological and Environmental Engineering, Chaohu University, Hefei, China; 2 School of Traditional Chinese Medicine, Wenzhou Medical University, Wenzhou, China; 3 Chaohu Regional Collaborative Technology Service Center for Rural Revitalization, Chaohu University, Hefei, China; 4 Yongkang First People’s Hospital of Wenzhou Medical University, Wenzhou Medical University, Wenzhou, China

**Keywords:** gene, inflammation, *Misgurnus anguillicaudatus*, *Polygonatum sibiricum* polysaccharides, transcriptome

## Abstract

*Misgurnus anguillicaudatus* (Cobitidae) is a small economic fish, which is widely distributed in ponds, reservoirs, and lakes in China. Because of its tasty meat and plentiful nutritional value, it is known as “water ginseng.” Saccharide is the most active component in the dry rhizome of the Chinese herbal medicine *Polygonatum sibiricum*, and the content of polysaccharide is the highest. It is reported that *Polygonatum sibiricum* polysaccharides (PSPs) have a significant therapeutic effect on inflammation. Based on the known pharmacological effects of PSPs, we fed PSPs to the acute inflammatory model of loach induced by lipopolysaccharides and analyzed the transcriptomic changes of the loach liver by RNA sequencing. The results show that a total of 10,250 differentially expressed genes (DEGs) were obtained after PSP treatment, and 5,652 DEGs were upregulated. The GO and KEGG enrichment analyses indicated that the DEGs regulated by PSPs are mainly involved in carbohydrate metabolism and the organismal immune system. After verifying the reliability of the sequencing results by RT-qPCR, we found that FcγR-mediated phagocytosis may be a key pathway for the anti-inflammatory effect of PSPs. This study provides important information on the molecular mechanism of PSPs in the treatment of inflammation in *M. anguillicaudatus*.

## 1 Introduction

Loach (*Misgurnus anguillicaudatus*) belongs to the genus *Misgurnus* of the family Cobitidae of the order Cypriniformes and is a benthic fish in warm water (Yang et al., 2021). As a small economic fish, loach is popular in many countries including China, Japan, Korea, Russia, and India (Yi et al., 2019; Wang et al., 2018). Because of its tender meat, rich nutritional value, and high protein content, loach is also known as “water ginseng” (Wang et al., 2022). Apart from that, it also has medicinal value. According to traditional Chinese medicine, loach has an effect of replenishing and benefiting qi, strengthening yang, and removing dampness–evil (The edible and medicinal value, 2009). Consuming loach that has been dried and ground into powder after removing the internal organs can improve the adverse symptoms of patients with acute hepatitis (The edible and medicinal value, 2009). At the same time, the liquid secreted by the loach skin also has antibacterial and anti-inflammatory effects. In China, loaches in the market are primarily obtained from artificial breeding rather than wild fishing. However, freshwater aquaculture of loach faces a series of threats. High-density farming methods have resulted in infectious diseases caused by pathogens including fungi, bacteria, and viruses (Yang et al., 2025). Vesicular disease, red speck disease, water mildew, rotten skin and rotten body, and enteritis are common diseases, and chemical drugs are usually used to control them (Yang et al., 2021). As is commonly known, long-term use of chemical drugs and antibiotics not only causes drug resistance to pathogens but also inhibits the host’s immune-protective ability. Because these infectious diseases cannot be effectively treated, it will lead to the large-scale death of loaches and cause a heavy loss to the operators (Zhang et al., 2023). Therefore, in order to maintain healthy and sustainable development of fisheries and ensure the economic benefits to farmers, it is very important to find effective alternative drugs to control the diseases related to loach farming.

Lipopolysaccharide (LPS) is a structural component of the cell wall of Gram-negative bacteria. It induces the secretion of a variety of inflammatory factors through the cell signal transduction system in the body, resulting in an inflammatory response (Wu et al., 2022). Therefore, we used an LPS-induced inflammatory model combined with transcriptomics technology to identify the key metabolic pathways in loach in response to the traditional Chinese medicine extract *Polygonatum sibiricum* polysaccharides (PSPs).

The traditional Chinese medicine Huangjing is the dried rhizome of *Polygonatum sibiricum*, *Polygonatum cyrtonema* Hua, and *Polygonatum kingianum*. They are perennial herbs of the genus *Polygonatum* in the Liliaceae family (Ali et al., 2020). Polysaccharides, saponins, and flavonoids are the main active substances of Huangjing, and polysaccharides are the most widely studied among them (Tang et al., 2019). The composition of PSPs is complex; monosaccharide composition analysis identified arabinose, glucose, rhamnose, galactose, ribose, fructose, xylose, and galacturonic acid, among others (Hu et al., 2023). These monosaccharides constitute different types of polysaccharides such as fructans and pectins (Cui et al., 2018; Zhao et al., 2020a; Zhao et al., 2020b). In conclusion, PSPs are a mixture of various types of polysaccharides. PSPs are a popular research subject because of their various pharmacological activities. Through *in vitro* and *in vivo* tests, Chen et al. demonstrated that PSPs play a positive immunomodulatory role in cellular and humoral immune functions (Chen et al., 2020). Zeng et al. showed that PSPs had good therapeutic effects on obesity in rats fed a high-fat diet, which can effectively reduce their body weight (Zeng et al., 2022). In addition, PSPs also have antidiabetic (Zeng et al., 2022), antioxidant (Li et al., 2021; Shen et al., 2021), and antidepressant (Shen et al., 2021) effects. Based on the effects of PSPs, we apply them to the loach breeding industry, which is expected to be a new method for the prevention and treatment of infectious diseases in loaches as it serves as an alternative to antibiotics.

Transcriptomic sequencing is widely used in the field of molecular biology, and it refers to the sequencing of cDNA using second-generation high-throughput sequencing technology, which in turn provides information about the transcripts of a particular sample comprehensively. In fact, RNA-seq for loach-related tissues is not the first analysis method reported: transcriptomic analysis of loach liver and testicular tissue has been reported earlier (Xu et al., 2023; Yang et al., 2022). For the purpose of exploring the anti-inflammatory mechanism of PSPs, we performed RNA-seq on the liver tissue of loach and used bioinformatics analysis techniques to mine anti-inflammatory-related genes. This established a foundation for elucidating the anti-inflammatory mechanism of PSPs in loach and provided new stratagems for the prevention and treatment of diseases in the loach breeding industry in the future.

## 2 Materials and methods

### 2.1 Animal sources and breeding conditions

The loaches used in the experiment were purchased from a seafood supermarket, Wenzhou City, Zhejiang Province, P.R. China, in July 2023. The loaches were moved to the laboratory of Wenzhou Medical University for 1 week and kept in a plastic rearing box with half volume of tap water. The water was changed once a day, and the rearing environment had good air circulation and a suitable temperature. They were fed with special feed for aquatic animals, and the feeding amount was 5% of the body weight of the loaches, provided twice a day.

### 2.2 Experimental design

The experiment was conducted in the laboratory of Wenzhou Medical University under natural conditions. On 9 July 2023, the loaches that were in good health and with consistent body shape were selected and randomly divided into two groups, with 20 individuals in each group. Healthy loaches were randomly divided into a control group (CK) and a treatment group (PSPs), with 20 fish per group, using a completely randomized design, in which the randomization sequence was generated by the RAND() function in Microsoft Excel. The PSPs group was pretreated with PSPs (PSPs mixed with feed in the ratio 1:2) for 12 h, and then each loach was injected with 0.2 mL LPS and continued to be fed with PSPs for 24 h. The CK group was fed with normal feed and injected with LPS at the same time as the PSP group. After 24 h, the CK and PSP groups of loaches were dissected; the liver tissue was taken, filled into EP tubes and frozen in liquid nitrogen for 15 min, and stored at −80 °C until the transcriptomic analysis (potential confounders were minimized through daily randomization of the experimental operation sequence and random assignment in the sampling process and personnel).

### 2.3 Transcriptomic profiling of *M*. *anguillicaudatus*

#### 2.3.1 RNA extraction, library construction, and sequencing analysis

Total RNA was extracted from loach liver tissues using TRIzol reagent (Invitrogen). RNA integrity was assessed by agarose gel electrophoresis, and RNA concentration and purity were measured using a NanoDrop spectrophotometer (Thermo Fisher Scientific). RNA samples with a total amount ≥1 μg were selected, and mRNA with a polyA tail was enriched using oligo(dT) magnetic beads. cDNA was synthesized using a strand-specific library preparation kit, and the purified double-stranded cDNA was then subjected to end repair, A-tailing, and adapter ligation. cDNA fragments of 400 bp–500 bp were selected using AMPure XP beads, followed by PCR amplification and bead purification to obtain the final cDNA library. After passing quality control, the library was paired-end-sequenced (PE150) on an Illumina platform (Liu et al., 2021). Raw reads were filtered using fastp (v 0.22.0) to remove adapters and low-quality sequences (Chen et al., 2018), thus yielding high-quality clean reads. The clean reads were assembled *de novo* using Trinity software (Grabherr et al., 2011) to obtain transcript sequences, and the assembled transcripts were then clustered, and redundant sequences were removed using Corset software (Davidson and Oshlack, 2014) to obtain unigene sequences.

#### 2.3.2 Identification and functional enrichment analysis of DEGs

The gene expression levels were quantified using RSEM software (Li and Dewey, 2011) and normalized by the fragments per kilobase of transcript per million mapped fragments (FPKM) method. Differentially expressed genes (DEGs) were identified using DESeq2 software (Love et al., 2014), with the false discovery rate (FDR) applied for multiple testing correction. The screening criteria were set as |log_2_ (fold change)| ≥ 1 and *P* < 0.05.

To explore the potential biological functions of the identified DEGs, Gene Ontology (GO) functional enrichment analysis and Kyoto Encyclopedia of Genes and Genomes (KEGG) pathway enrichment analysis were performed. For GO enrichment analysis, the DEGs were mapped to the GO database, and significantly enriched GO terms in the three categories (biological process, cellular component, and molecular function) were identified using the hypergeometric distribution test (*P* < 0.05). KEGG pathway enrichment analysis was performed using the same method (hypergeometric distribution test) to systematically identify the metabolic and signaling pathways that were significantly enriched by the DEGs (*P* < 0.05), thereby elucidating their potential biological roles at the pathway level.

### 2.4 RT-qPCR analysis of DEGs

A total of 13 DEGs were randomly selected for validation, and β-actin was used as the housekeeping gene (Kuang et al., 2024). Primer design was performed using Lasergene 7.1.0 software, and the primer sequences are shown in Supplementary Table S1. The primers were subsequently synthesized by Shenggong Bioengineering Co., Ltd (Shanghai, China). Total RNA was extracted from loach liver for the CK and PSP groups, respectively, in the laboratory using an animal RNA kit (RNA Easy Fast, TIANGEN). After the total RNA was quality-checked, cDNA was synthesized according to the TransScript® One-Step cDNA Synthesis SuperMix (TransGen) instructions. Both cDNAs were quantified at 500 ng/uL, and the systems were mixed according to the instructions for PerfectStart® Green qPCR SuperMix (TransGen). The total system volume was 20 uL, including 1 uL of the template, 0.5 uL each of the forward and reverse primer, 10 uL of 2 × PerfectStart® Green qPCR SuperMix, and 7.5 uL of enzyme-free water. Subsequently detected using a QuantStudio™ 6 Flex real-time fluorescence quantitative PCR instrument (ABI). RT-qPCR was performed using a three-step method: denaturation at 94 °C for 5 s, annealing temperature with reference to the Tm value of the primers and time for 30 s, extension at 72 °C for 10 s, and the number of cycles set to 40. Finally, the gene was quantitatively analyzed using the 2^−ΔΔCt^ method.

## 3 Results

### 3.1 RNA-seq and transcriptomic analysis

**FIGURE 1:**
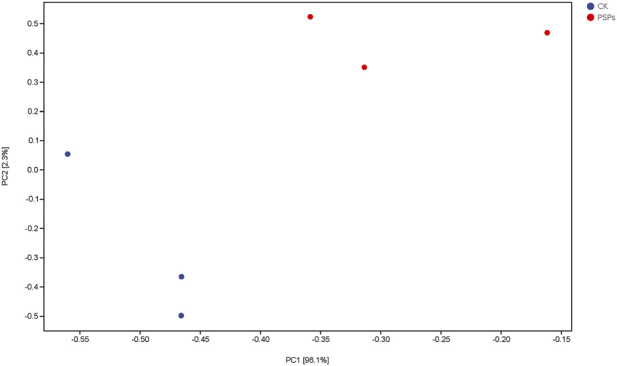
Principal component analysis (PCA) of the CK and PSP groups. Each group contained three biological replicate samples, and each replicate sample was prepared from a pool of six loaches. Each data point represents one pooled biological replicate.

For the purpose of exploring the effect of PSPs on loach inflammation, we sequenced the samples under PSP treatment. After filtering the raw data from the CK and PSP libraries, we obtained between 39.71 and 48.33 million clean reads per library. The percentage of high-quality sequence reads in the sequenced reads is greater than 97.87% (Supplementary Table S2). After quality assessment, the remaining high-quality clean reads were merged and assembled, and a total of 243,533 transcripts and 94,772 unigenes were obtained (Table 1). Among them, the maximum length of the unigenes was 48,863 bp, the average length was 1,015.57 bp, and the total length was 96,247,359 bp. We performed principal component analyses (PCAs) based on the expression of genes in the CK and PSP groups, thus analyzing intra- versus inter-group differences (Figure 1). It can be seen that at the PC1 level (96.1%), the two samples are farther apart, indicating significant genotypic differences between CK and PSP groups. At the PC2 level (2.3%), the intra-group differences between the CK and PSP groups were large, which may be caused by individual differences.

**TABLE 1:** Statistical results of transcript and unigene sequences.

Item	Transcript	Unigene
Total length (bp)	315,438,213	96,247,359
Sequence number	243,533	94,772
Max. length (bp)	48,863	48,863
Mean length (bp)	1,295.26	1,015.57
GC%	42.21	41.37

Total length (bp): total length of sequence. Sequence number: total number of sequences. Max. length (bp): maximum length of sequence. Mean length (bp): average length of sequence. GC%: GC content of the sequence.

A total of 68,168 unigenes were functionally annotated in six public databases: NR, Swiss-Prot, KEGG, GO, eggNOG, and Pfam (Table 2). The results indicated that 32,523 unigene sequences (47.71%) were significantly annotated in the NR database, with the best hit rate. In comparison, the unigenes which were successfully annotated to the Swiss-Prot, KEGG, GO, eggNOG, and Pfam databases were 24,944 (36.59%), 8,916 (13.08%), 21,622 (31.72%), 29,972 (43.97%), and 21,250 (31.17%) entries, respectively. Finally, only 5,453 unigene sequences (9.54%) had mapped against in all six databases.

**TABLE 2:** Success rate of gene annotation.

Database	Number	Percentage (%)
NR	32,523	47.71
GO	21,622	31.72
KEGG	8,916	13.08
Pfam	21,250	31.17
eggKOG	29,972	43.97
Swiss-Prot	24,944	36.59
In all database	6,502	9.54

GO: the unigene number and annotation rate in the GO database. KEGG: the unigene number and annotation rate in the KEGG database. eggNOG: the unigene number and annotation rate in the eggNOG database. Pfam: the unigene number and annotation rate in the Pfam database. NR: the unigene number and annotation rate in the NR database. Swiss-Prot: the unigene number and annotation rate in the Swiss-Prot database. In all database: the unigene number annotated in all databases.

### 3.2 Analysis of DEGs and KEGG pathway enrichment analysis

**FIGURE 2:**
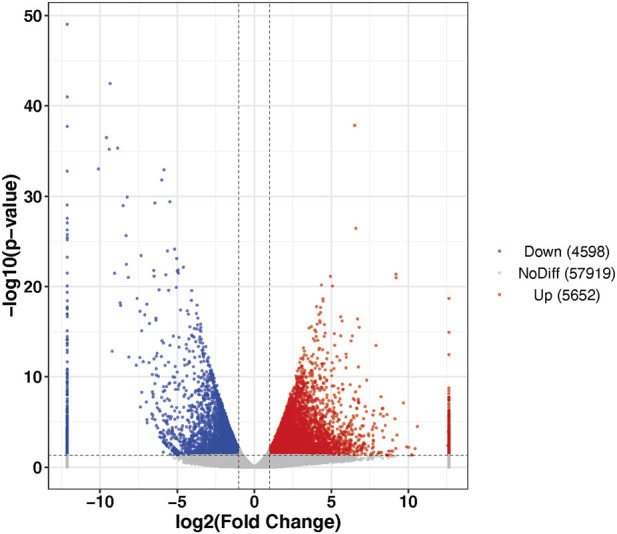
Differential gene volcano map in the CK vs. PSP groups.

**FIGURE 3:**
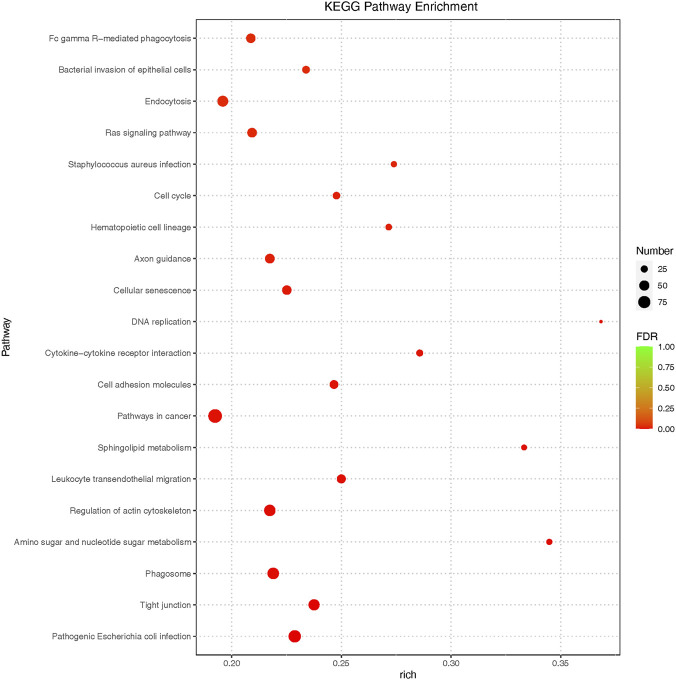
Top-20 KEGG pathways enriched in differentially expressed genes between the CK and PSP groups.

A total of 10,250 DEGs were identified based on the screening threshold value between the CK and PSP groups (Figure 2). Compared with the number of downregulated genes, the number of upregulated genes was higher, 5,652 and 4,598, respectively. To determine which biological pathways these DEGs are involved in, they were subjected to KEGG pathway analysis. We found that the 5,652 upregulated DEGs were enriched in 337 pathways. Figure 3 shows the top-20 significantly enriched pathways (Supplementary Table S3). They are enriched in a variety of different pathways, among which the immune system-related pathways are leukocyte trans-endothelial migration, hematopoietic cell lines, and Fc gamma R-mediated phagocytosis. In addition, regulation of the actin cytoskeleton belongs to the category of cell motility in cellular processes, but it is also closely related to the function of a wide range of immune cells. The anti-inflammatory effect of PSPs is closely related to the regulation of immune response-related pathways. The upregulated genes in these immune-related pathways have a certain effect on the disease resistance in loaches.

### 3.3 Functional classification of DEGs using the GO database

**FIGURE 4:**
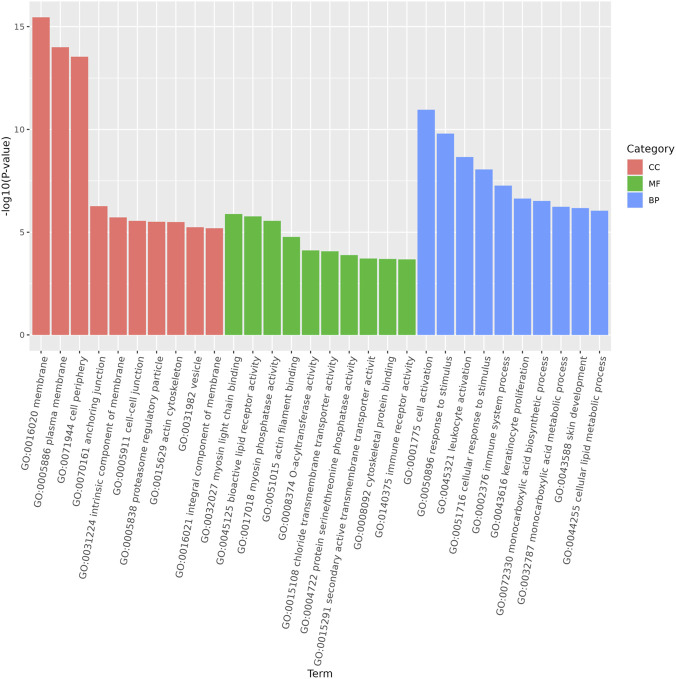
GO classification of differentially expressed genes in the CK vs. PSP groups.

To understand the functional role of EDGs, they were aligned to the GO database. Figure 4 shows the top-10 terms that are significantly enriched in each category. In the biological process category, a multitude of DEGs were annotated under “cell activation” (GO:0001775), “response to stimulus” (GO:0050896), “leukocyte activation” (GO:0045321), “cellular response to stimulus” (GO:0051716), “immune system process” (GO:0002376), “keratinocyte proliferation” (GO:0043616), and “monocarboxylic acid biosynthetic process” (GO:0072330) under PSP treatment. It indicated that PSPs can enhance the immune response process in loaches. We found that in the molecular function category, “myosin light chain binding” (GO:0032027) and “bioactive lipid receptor activity” (GO:0032027) were the most highly enriched GO terms. The DEGs of the cellular components category were enriched in “membrane” (GO: 0016020) and “plasma membrane” (GO: 0005886), suggesting that PSPs lead to increased metabolic activity on the cell membrane. These results reveal that PSP may regulate the activity of cell metabolism and immune response.

### 3.4 Gene validation of RNA-seq by RT-qPCR

**FIGURE 5:**
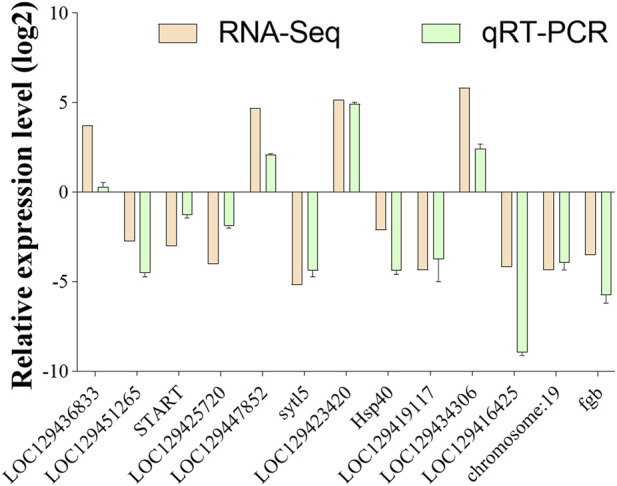
Verification of the reliability of RNA-seq data using RT-qPCR.

The expression of the 13 DEGs that were screened was verified by real-time fluorescence quantitative PCR, and the results of RT-qPCR indicated that the expressions of four genes were upregulated and those of nine genes were downregulated. This is in agreement with the RNA-seq results, as shown in Figure 5. This indicates that the sequencing data are greatly reliable and the subsequent correlation analyses are based on actual transcriptomic changes.

## 4 Discussion

### 4.1 Analysis of the therapeutic effect of PSPs in loaches

Pharmacological studies have indicated that PSPs have anti-inflammatory, antitumor, and antioxidant biological activities (Sun et al., 2023), and these effects have a good preventive and therapeutic effect on inflammation in loaches. Cui et al. found that PSP protects chondrocytes and alleviates osteoarthritis by inhibiting the TLR2/NF-κB signaling pathway (Kuang et al., 2024). Jiang et al. demonstrated that PSP alleviates cognitive impairment by regulating gut microbiota, reducing intestinal permeability, and lowering serum endotoxin levels (Jiang et al., 2024). Furthermore, the therapeutic potential of plant-derived polysaccharides has also been confirmed in other aquatic animals. Luo et al. showed that dietary supplementation with ginger polysaccharide significantly improved the growth performance, serum lysozyme activity, and antioxidant capacity of crucian carp and reduced the mortality rate after *Aeromonas hydrophila* infection (Luo et al., 2024). Pei et al. found that *Salvia miltiorrhiza* polysaccharide promotes the health of crayfish by enhancing hemocyte phagocytosis, protecting the hepatopancreas, and strengthening the intestinal barrier function (Pei et al., 2024). Meanwhile, Kong et al. comprehensively reviewed the immunomodulatory, antioxidant, and antibacterial properties of β-glucan in aquaculture, emphasizing that plant polysaccharides are sustainable alternatives to antibiotics (Kong et al., 2025). Therefore, in order to investigate the role of PSPs in the outbreak of inflammation in aquatic animals (e.g., loach), in this study, we established a loach model of LPS-induced inflammation, and the concentration of PSPs added to the feed was confirmed to be non-toxic to the loach in the previous experiments. The mortality rate of loach was 50% in the CK group and 20% in the PSPs group within 24 h after LPS injection, which shows that PSPs have a therapeutic effect on inflammation in loaches. The potential anti-inflammatory mechanism of PSPs can be explored at the molecular level by transcriptomic analysis. The results showed that the upregulated genes were significantly enriched in amino sugar and nucleotide sugar metabolism, FcγR-mediated phagocytosis, leukocyte trans-endothelial migration, sphingolipid metabolism, and other metabolic pathways. These pathways are key pathways for PSPs to exert anti-inflammatory effects, and the subsequent analysis of these pathways helps mine candidate genes for the anti-inflammatory effects.

### 4.2 Analysis of DEGs regulating immune-related signaling pathways

Through the enrichment analysis of upregulated DEGs, we found that the FcγR-mediated phagocytosis signaling pathway was significantly enriched. There are 43 EDGs that regulate this pathway, and four EDGs are specifically expressed (Supplementary Table S4). FcγR-mediated phagocytosis is an important component of the body’s immune system, which can effectively remove pathogens and other exogenous substances. Fcγ receptor (FcγR) is the Fc segment receptor of immunoglobulin G (lgG) and is mainly expressed on the immune cell membrane, such as macrophages, monocytes, and neutrophils. FcγR has three categories: FcγRI, FcγRII (FcγRIIA, FcγRIIB, and FcγRIIC), and FcγRIII (FcγR IIIA and FcγR IIIB) (Cui et al., 2018). Varsha et al. demonstrated that during the macrophage activation syndrome, miRNAs target the FcγR-mediated phagocytosis pathway and upregulate the expressions of Fcgr3, Fcgr4, and Fcgr1 genes, thereby enhancing macrophage phagocytosis (Varsha et al., 2024). Bond et al. found that the Fc receptors prime macrophages for increased sensitivity to IgG through both short-term and long-term mechanisms, thereby improving antibody-dependent phagocytosis (Bond et al., 2024).

**FIGURE 6:**
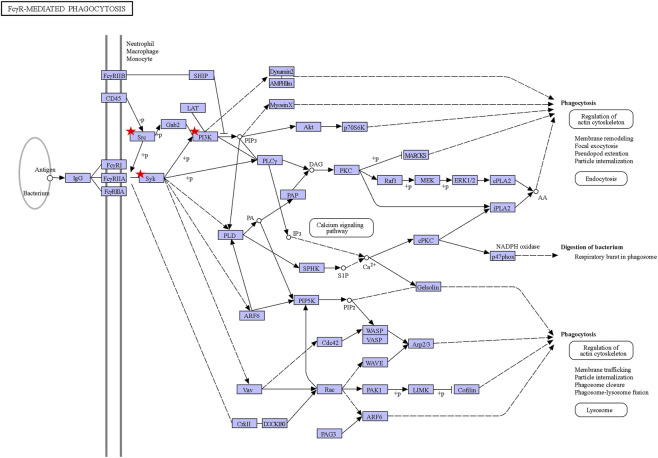
Overview of the FcγR-mediated phagocytosis pathway.

Figure 6 shows that when the antigen is bound by IgG specifically, the FcγRs on the cell membrane recognize the antibody, followed by Src kinase recruiting ITAM and causing tyrosine of ITAM phosphorylation, which is the triggering signal of FcγR-mediated phagocytosis. Afterward, Syk kinase is recruited to phosphorylated ITAM, and Syk kinase is activated, which can directly excite PI3K and PLCγ proteins and indirectly affect other downstream proteins. By triggering the activation of multiple pathways, these pathogen-carrying IgG are eventually phagocytosed by macrophages. Syk kinase and Src kinase play a critical role in FcγR-mediated phagocytosis because FcRIIA-mediated phagocytosis is attenuated or completely inhibited in cells where Src kinase and Syk kinase are not expressed (Suzuki et al., 1998). On the other hand, actin cytoskeleton remodeling has an important effect on the phagocytosis of macrophages, which can promote the chemotaxis of macrophages to the inflammatory response at the site of infection (Strzelecka-Kiliszek et al., 2002). The conduction of PI3K-Akt in the FcγR-mediated phagocytosis signaling pathway has a close relationship with the regulation of the actin cytoskeleton (Ronzier et al., 2022).

**FIGURE 7:**
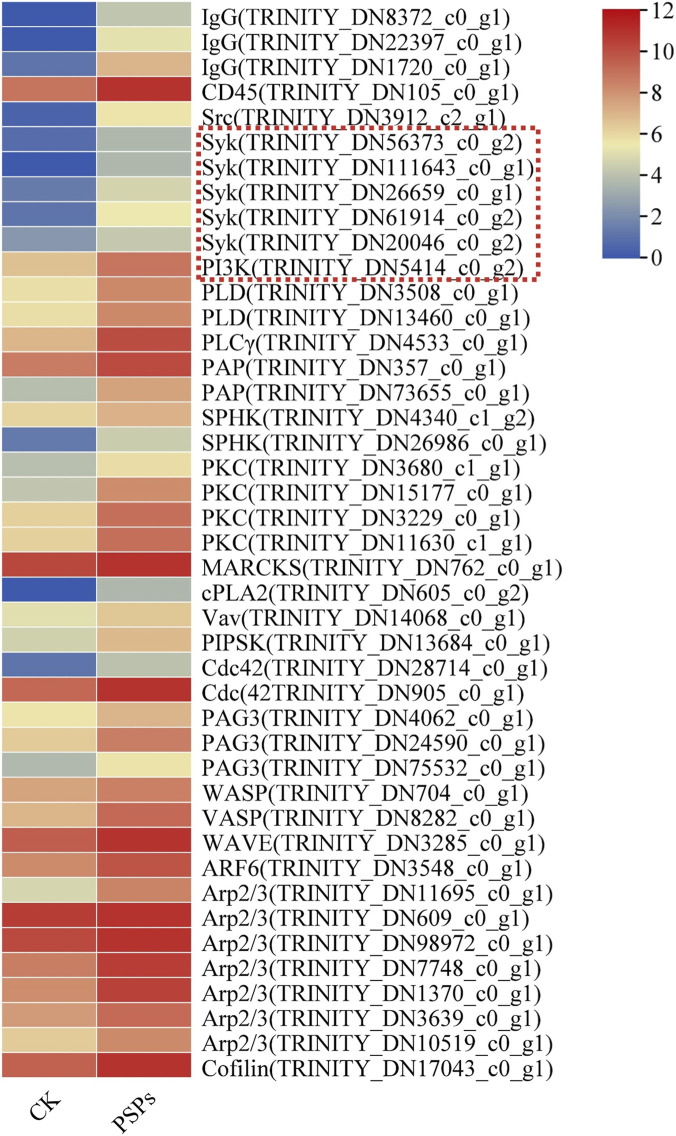
Heatmap of 43 differential gene expressions. The expression value of genes was calculated by log2 transformation of the FPKM-normalized counts.

The EDG analysis of this pathway showed that (Figure 7) the Src kinase family, Syk kinase family, and PI3K kinase family were regulated by one, five, and one genes, respectively. Among others, TRINITY_DN111643_c0_g1 is a gene specifically expressed after PSP treatment, and the gene TRINITY_DN3912_c2_g1 has the greatest difference in expression. These seven upregulated DEGs are likely to be candidate genes for enhancing FcγR-mediated phagocytosis. The potential molecular mechanism of the anti-inflammatory effects of PSPs needs to be further confirmed by functional verification analysis of these candidate genes.

## 5 Conclusion

The present study indicates that the therapeutic effect of PSPs on loaches with LPS-induced inflammation may be achieved by enhancing the FcγR-mediated phagocytosis signaling pathway. Pre-feeding of PSPs improved the immunity of loaches. Therefore, when inflammation occurred, the genes responding to PSPs were highly expressed to enhance the immune response-related signaling pathways, thereby improving the body’s disease resistance. Enrichment analysis of DEGs led us to believe that the FcγR-mediated phagocytosis signaling pathway plays a key role in the immune response of loaches, which was confirmed by the increased expression levels of genes encoding enzymes and various proteins in the pathway. We also found that genes encoding Src, Syk, and PI3K proteins may be key genes, and further studies should be carried out to confirm their functions. These results will help reveal the molecular mechanism by which PSPs exert anti-inflammatory effects in the diseases of loach culture, contribute to the development of antibiotic alternatives in the future, and provide a new insight into the construction of a healthy fish-breeding industry.

## Data Availability

The datasets presented in this study can be found in online repositories. The names of the repository/repositories and accession number(s) can be found below: https://www.ncbi.nlm.nih.gov/genbank/, SRR29839230.
