# Supplementary material for: Transcriptomic analysis reveals FcγR-mediated phagocytosis as a key pathway for the anti-inflammatory action of Polygonatum sibiricum polysaccharides in loach
Source: Front Genet. 2026 Jun 19;17:1733253. doi: 10.3389/fgene.2026.1733253 (PMC13327655; doi:10.3389/fgene.2026.1733253)
Supplement: Supplementary file 1 [file Table1.doc]

**Table S1 The genes and primers used for qRT-PCR analysis**

| Gene name | Primer name | Sequence (5’-3’) |
| --- | --- | --- |
| *LOC129436833* | 6458-F | CCATCCGGATCGCATTAT |
|  | 6458-R | AACCCCAGCTTGTCATTTTT |
| *LOC129451265* | 1380-F | TGGCCCGTCTCCTATCAT |
|  | 1380-R | TGTAAATTCCCTGCTGTAACCA |
| *START* | 41427-F | CCCACCAGCGCCAGCAGTCTA |
|  | 41427-R | GGTGTCTTCGGGCGGTGTCG |
| *LOC129425720* | 577-F | GGCTCGCCCCCATCTGCT |
|  | 577-R | TGGAGGGCTTAGGACGAGGAGT |
| *LOC129423420* | 292-F | GTCCCGAATCTGCCAACCTA |
|  | 292-R | GCTCACGGGAACCAATCAAG |
| *LOC129447852* | 1422-F | GCTGGGGTGCATTCCTAC |
|  | 1422-R | GGGGCCATACTTTGCTGTTT |
| *sytl5* | 86769-F | AGGCGTCGAGCTCTTGATGA |
|  | 86769-R | TTTAGCTGCTGCCACCCTGAG |
| *LOC129434306* | 1547-F | CAGCGTGAAAACCAGCGTATTA |
|  | 1547-R | GCCCCAAGCACCAAAACT |
| *Hsp40* | 6030-F | GAAGGGGTGTGCAGGTCCAG |
|  | 6030-R | TTGCGTTCTACTTTGCGTCCAT |
| *LOC129419117* | 33681-F | CGAGCGCTTTGATGTGGAGGTA |
|  | 33681-R | TCAGCAGCGAAAATAGGAGAGG |
| *chromosome: 19* | 16401-F | CCCCTCGCCCCGTTACCAC |
|  | 16401-R | CCGGGCAGGAATCCAGCATCT |
| *LOC129416425* | 10593-F | AGCCCATGTACCCCGAGCAG |
|  | 10593-R | TTTTCCCGCCACATTTTACACA |

**Table S1 continued**

| Gene name | Primer name | Sequence (5’-3’) |
| --- | --- | --- |
| *fgb* | 2013-F | ATCCGCTCAACATGCCTTACAA |
|  | 2013-R | AGCGCCTGCCAAAATCAAC |
| *β-actin* | actin-F | AGAGAGAAATTGTCCGTGAC |
|  | actin-R | GCCAATGGTGATGACCTGT |

**Table S2 Clean data statistics**

| **Sample** | **Clean Reads** | **Clean Data (bp)** | **Clean Reads%** | **Clean Data%** |
| --- | --- | --- | --- | --- |
| CK_1 | 48332086 | 7282804437 | 98.67 | 98.46 |
| CK_2 | 40208890 | 6051949500 | 97.87 | 97.56 |
| CK_3 | 46989740 | 7084077034 | 98.83 | 98.67 |
| PSPs_1 | 44314948 | 6679491970 | 98.69 | 98.52 |
| PSPs_2 | 46797282 | 7051586871 | 98.29 | 98.08 |
| PSPs_3 | 39715114 | 5984894561 | 98.71 | 98.51 |

Sample: description of sample, Clean Reads: high quality sequence read number, Clean Data(bp): high quality

sequence base number, Clean Reads%: the percentage of high-quality sequence reads in sequencing reads,

Clean Data%: The percentage of high-quality sequence bases in sequencing bases.

.

**Table S3 The top 20 KEGG pathways enriched in DEGs**

| PathwayID | Pathway | Level1 | Level2 | list_number | total_number | Pvalue | FDR |
| --- | --- | --- | --- | --- | --- | --- | --- |
| ko05130 | Pathogenic Escherichia coli infection | Human Diseases | Infectious disease: bacterial | 78 | 341 | 9.56658E-07 | 0.000322394 |
| ko04530 | Tight junction | Cellular Processes | Cellular community - eukaryotes | 62 | 261 | 3.41524E-06 | 0.000575468 |
| ko04145 | Phagosome | Cellular Processes | Transport and catabolism | 67 | 306 | 2.54425E-05 | 0.002846944 |
| ko00520 | Amino sugar and nucleotide sugar metabolism | Metabolism | Carbohydrate metabolism | 20 | 58 | 3.62991E-05 | 0.002846944 |
| ko04810 | Regulation of actin cytoskeleton | Cellular Processes | Cell motility | 65 | 299 | 4.22395E-05 | 0.002846944 |
| ko04670 | Leukocyte transendothelial migration | Organismal Systems | Immune system | 39 | 156 | 6.69473E-05 | 0.003760209 |
| ko00600 | Sphingolipid metabolism | Metabolism | Lipid metabolism | 19 | 57 | 9.56895E-05 | 0.004606767 |
| ko05200 | Pathways in cancer | Human Diseases | Cancer: overview | 97 | 504 | 0.000113718 | 0.004790386 |
| ko04514 | Cell adhesion molecules | Environmental Information Processing | Signaling molecules and interaction | 37 | 150 | 0.000137221 | 0.005138158 |
| ko04060 | Cytokine-cytokine receptor interaction | Environmental Information Processing | Signaling molecules and interaction | 24 | 84 | 0.000194363 | 0.006550037 |
| ko03030 | DNA replication | Genetic Information Processing | Replication and repair | 14 | 38 | 0.000240173 | 0.007358041 |

**Table S3 continued**

| PathwayID | Pathway | Level1 | Level2 | list_number | total_number | Pvalue | FDR |
| --- | --- | --- | --- | --- | --- | --- | --- |
| ko04218 | Cellular senescence | Cellular Processes | Cell growth and death | 43 | 191 | 0.00036892 | 0.010360507 |
| ko04360 | Axon guidance | Organismal Systems | Development and regeneration | 45 | 207 | 0.000611343 | 0.01584789 |
| ko04640 | Hematopoietic cell lineage | Organismal Systems | Immune system | 22 | 81 | 0.000759953 | 0.01776971 |
| ko04110 | Cell cycle | Cellular Processes | Cell growth and death | 28 | 113 | 0.000790937 | 0.01776971 |
| ko05150 | Staphylococcus aureus infection | Human Diseases | Infectious disease: bacterial | 20 | 73 | 0.00115343 | 0.024294124 |
| ko04014 | Ras signaling pathway | Environ Cofilin mental Information Processing | Signal transduction | 45 | 215 | 0.001401019 | 0.027773145 |
| ko04144 | Endocytosis | Cellular Processes | Transport and catabolism | 58 | 296 | 0.001689514 | 0.030246409 |
| ko05100 | Bacterial invasion of epithelial cells | Human Diseases | Infectious disease: bacterial | 29 | 124 | 0.001705287 | 0.030246409 |
| ko04666 | Fc gamma R-mediated phagocytosis | Organismal Systems | Immune system | 43 | 206 | 0.001867068 | 0.031460092 |

**Table S4 Gene expression in FcγR-mediated phagocytosis pathway**

| Gene ID | Description | CK(fpkm) | PSPs(fpkm) |
| --- | --- | --- | --- |
| TRINITY_DN8372_c0_g1 | IgG | 0 | 16.59652304 |
| TRINITY_DN22397_c0_g1 | IgG | 0 | 36.09426489 |
| TRINITY_DN1720_c0_g1 | IgG | 1.140499448 | 127.8258242 |
| TRINITY_DN105_c0_g1 | CD45 | 472.8257409 | 2230.961616 |
| TRINITY_DN3912_c2_g1 | Src | 0.384637017 | 47.94475348 |
| TRINITY_DN56373_c0_g2 | Syk | 0.769274033 | 11.61196896 |
| TRINITY_DN111643_c0_g1 | Syk | 0 | 11.54714845 |
| TRINITY_DN26659_c0_g1 | Syk | 1.66976587 | 25.14407705 |
| TRINITY_DN61914_c0_g2 | Syk | 1.144969982 | 42.61006751 |
| TRINITY_DN20046_c0_g2 | Syk | 4.393594048 | 18.52504333 |
| TRINITY_DN5414_c0_g2 | PI3K | 98.90834273 | 464.4948741 |
| TRINITY_DN3508_c0_g1 | PLD | 53.83815117 | 313.376843 |
| TRINITY_DN13460_c0_g1 | PLD | 53.83815117 | 313.376843 |
| TRINITY_DN4533_c0_g1 | PLCγ | 127.4509818 | 1099.129839 |
| TRINITY_DN357_c0_g1 | PAP | 408.7761824 | 1168.912526 |
| TRINITY_DN73655_c0_g1 | PAP | 13.72033598 | 180.6642942 |
| TRINITY_DN4340_c1_g2 | SPHK | 67.31258733 | 134.7082965 |
| TRINITY_DN26986_c0_g1 | SPHK | 1.429758258 | 20.51632566 |
| TRINITY_DN3680_c1_g1 | PKC | 13.89633445 | 55.80832011 |
| TRINITY_DN15177_c0_g1 | PKC | 17.29987533 | 286.2101346 |
| TRINITY_DN3229_c0_g1 | PKC | 72.11289022 | 530.7267995 |
| TRINITY_DN11630_c1_g1 | PKC | 72.11289022 | 530.7267995 |
| TRINITY_DN762_c0_g1 | MARCKS | 1252.68371 | 3464.016712 |

**Table S4 continued**

| Gene ID | Description | CK(fpkm) | PSPs(fpkm) |
| --- | --- | --- | --- |
| TRINITY_DN605_c0_g2 | cPLA2 | 0 | 11.57874095 |
| TRINITY_DN14068_c0_g1 | Vav | 33.55648259 | 87.46384881 |
| TRINITY_DN13684_c0_g1 | PIPSK | 23.51233519 | 115.4059346 |
| TRINITY_DN28714_c0_g1 | Cdc42 | 1.15391105 | 14.85074912 |
| TRINITY_DN905_c0_g1 | Cdc42 | 618.3980969 | 2739.62052 |
| TRINITY_DN4062_c0_g1 | PAG3 | 48.92415644 | 128.0381347 |
| TRINITY_DN24590_c0_g1 | PAG3 | 82.1971971 | 397.1890838 |
| TRINITY_DN75532_c0_g1 | PAG3 | 11.93149289 | 50.5579811 |
| TRINITY_DN704_c0_g1 | WASP | 175.2731711 | 376.8312157 |
| TRINITY_DN8282_c0_g1 | WASP | 126.5531074 | 611.9898408 |
| TRINITY_DN3285_c0_g1 | WAVE | 764.8593109 | 1921.798894 |
| TRINITY_DN3548_c0_g1 | ARF6 | 291.1894377 | 945.5988113 |
| TRINITY_DN11695_c0_g1 | Arp2/3 | 26.29890149 | 345.4609773 |
| TRINITY_DN609_c0_g1 | Arp2/3 | 1590.609422 | 3932.105055 |
| TRINITY_DN98972_c0_g1 | Arp2/3 | 1186.044155 | 4037.601653 |
| TRINITY_DN7748_c0_g1 | Arp2/3 | 391.1243621 | 1515.108264 |
| TRINITY_DN1370_c0_g1 | Arp2/3 | 285.8683779 | 1402.688658 |
| TRINITY_DN3639_c0_g1 | Arp2/3 | 216.2677791 | 581.0282818 |
| TRINITY_DN10519_c0_g1 | Arp2/3 | 80.92555518 | 300.7704971 |
| TRINITY_DN17043_c0_g1 | Cofilin | 695.935332 | 3391.068751 |
